# Synphilin-1 Interacts with AMPK and Increases AMPK Phosphorylation

**DOI:** 10.3390/ijms21124352

**Published:** 2020-06-18

**Authors:** Tianxia Li, Jingnan Liu, Gongbo Guo, Bo Ning, Xueping Li, Guangjing Zhu, Dejun Yang, Timothy H. Moran, Wanli W. Smith

**Affiliations:** 1 Department of Psychiatry, Johns Hopkins University School of Medicine, Baltimore, MD 21205, USA; Tianxia_li@dfci.harvard.edu (T.L.); gguo1@jhmi.edu (G.G.); bning2@jhmi.edu (B.N.); xuepingli457@gmail.com (X.L.); guangjingzhu@hotmail.com (G.Z.); yangdjfeeding@gmail.com (D.Y.); tmoran@jhmi.edu (T.H.M.); 2 Department of Pharmaceutical Sciences, University of Maryland School of Pharmacy, Baltimore, MD 21201, USA; liujn@shanghaitech.edu.cn; 3 Institute of Obesity and Metabolic Diseases, School of Clinical Medicine, Xi’an Medical Collage, Xi’an 71000, China

**Keywords:** synphilin-1, AMPK, ATP, energy balance, protein interaction

## Abstract

A role for the cytoplasmic protein synphilin-1 in regulating energy balance has been demonstrated recently. Expression of synphilin-1 increases ATP levels in cultured cells. However, the mechanism by which synphilin-1 alters cellular energy status is unknown. Here, we used cell models and biochemical approaches to investigate the cellular functions of synphilin-1 on the AMP-activated protein kinase (AMPK) signaling pathway, which may affect energy balance. Overexpression of synphilin-1 increased AMPK phosphorylation (activation). Moreover, synphilin-1 interacted with AMPK by co-immunoprecipitation and GST (glutathione S-transferase) pull-down assays. Knockdown of synphilin-1 reduced AMPK phosphorylation. Overexpression of synphilin-1 also altered AMPK downstream signaling, i.e., a decrease in acetyl CoA carboxylase (ACC) phosphorylation, and an increase in p70S6K phosphorylation. Treatment of compound C (an AMPK inhibitor) reduced synphilin-1 binding with AMPK. In addition, compound C diminished synphilin-1-induced AMPK phosphorylation, and the increase in cellular ATP (adenosine triphosphate) levels. Our results demonstrated that synphilin-1 couples with AMPK, and they exert mutual effects on each other to regulate cellular energy status. These findings not only identify novel cellular actions of synphilin-1, but also provide new insights into the roles of synphilin-1 in regulating energy currency, ATP.

## 1. Introduction

Recent studies in transgenic animal models suggest that synphilin-1 is involved in regulating energy homeostasis [1,2]. Synphilin-1, a 919-amino acid protein, is expressed in various tissues and has high expression in the brain [3,4,5]. The full range of biological functions of synphilin-1 is not clear. Previous studies have shown that synphilin-1 can interact with multiple proteins (e.g., alpha-synuclein) and is involved in the pathogenesis of Parkinson’s disease (PD) in relation to protein aggregation [3,4,6,7,8,9,10,11,12,13,14], suggesting a putative role of synphilin-1 in PD. We and others have demonstrated that synphilin-1 has neurotrophic and neuroprotective effects [15,16,17,18,19]. The recent studies of human synphilin-1 in transgenic flies and mice have demonstrated that synphilin-1 induces hyperphagia and obesity [1,2]. In cultured cells, expression of synphilin-1 increased cellular ATP levels [2]. However, it is not clear how synphilin-1 alters the energy balance.

AMP-activated protein kinase (AMPK, a serine/threonine kinase) is an integrator of regulatory signals that modulate the systemic and cellular energy status in response to environmental nutritional changes [20,21,22,23,24,25]. Disruption of the AMPK pathway leads to metabolic dysregulation, resulting in various metabolic disorders. AMPK regulates a wide range of metabolic pathways [26,27] and has become a therapeutic target for the treatment of type II diabetes mellitus and obesity [27]. In the hypothalamus, AMPK is a master regulator of energy homeostasis by integrating nutrient and hormonal signals [24,28]. A decrease in hypothalamic AMPK activity is associated with suppression of feeding behavior, while activation of AMPK leads to increased food intake. Insulin and leptin suppress hypothalamic AMPK activity [29]. Orexigenic peptides such as the gut-derived hormone ghrelin and the neuropeptide agouti-related peptide [29] stimulate hypothalamic AMPK activity to increase food intake [30,31].

AMPK and synphilin-1 are both predominantly expressed in the cytosol. However, whether synphilin-1 and AMPK affect one another in controlling energy balance is not known. Thus, in this study, we used molecular biology tools and biochemical approaches to explore the relationship and functional consequences of the interactions between synphilin-1 and AMPK. Our findings identified a novel interaction between synphilin-1 and AMPK, which regulates cellular ATP levels. Therefore, we conclude that synphilin-1 interacts with AMPK and modulates energy homeostasis in cells.

## 2. Results

### 2.1. Synphilin-1 Increased AMPK Phosphorylation

**Figure 1:**
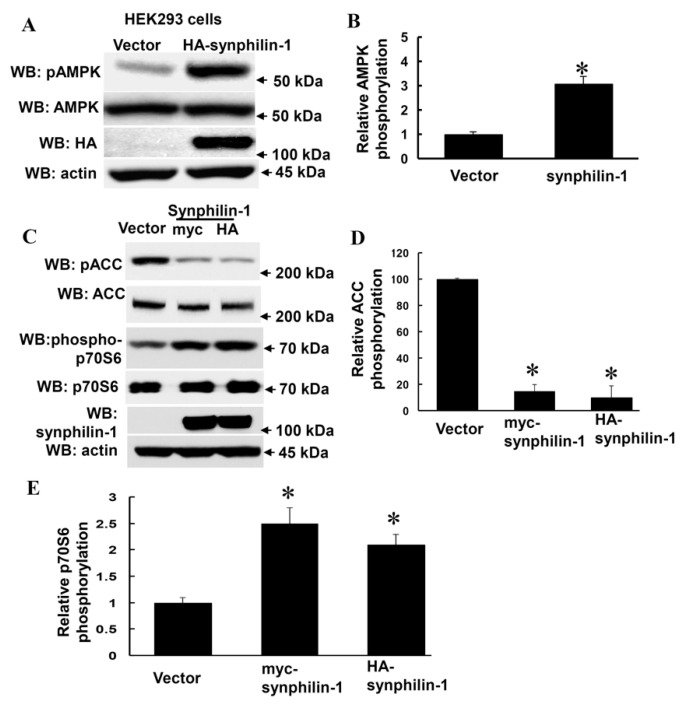
Overexpression of Synphilin-1 increased AMPK phosphorylation. (**A**) HEK293 cells were transfected with myc-synphilin-1 and vector for 48 h. (**A**,**B**) Cell lysates from HEK293 cells expressing human synphilin-1 were subjected to immunoblot analysis using anti-HA, anti-AMPKα, anti-phosphorylation AMPKα (pAMPK), and anti-actin antibodies. (**A**). Representative blots from three separated experiments. (**B**) Quantification of phosphorylated AMPKα levels normalized to total AMPKα levels. (**C**–**E**) Cell lysates from HEK293 cells expressing human synphilin-1 were subjected to immunoblot analysis using anti-ACCα, anti-phospho-ACCα (pACC), anti-phospho-p70S6, anti-p70S6, anti-synphilin-1, and anti-actin antibodies. (**C**) Representative blots from three separated experiments. (**D**,**E**). Quantification of phosphorylated ACCα and p70S6 levels normalized to total ACCα and p70S6 levels. * *p* < 0.05 by ANOVA followed by Tukey’s post-hoc test, vs. vector control cells.

Increased AMPKα phosphorylation at threonine 172 reflects increased AMPK activation [32]. To assess whether synphilin-1 alters AMPK phosphorylation, HEK 293 cells were transiently transfected with human synphilin-1, followed by a Western blot analysis using anti-phospho-AMPKα antibodies (at the threonine residue 172). Expression of human synphilin-1 significantly increased AMPK phosphorylation at threonine residue 172, up to ~3 fold (Figure 1A,B) compared with vector cells.

Moreover, synphilin-1 also altered AMPK downstream targets, i.e., it decreased acetyl CoA carboxylase (ACC) phosphorylation (Figure 1C,D), and increased p70 ribosomal S6 kinase (p70S6K) phosphorylation (Figure 1C,E). There were no significant differences detected in total AMPKα, ACC and p70S6K levels between cells expressing synphilin-1 and just the vector (Figure 1A,C). These results suggest that synphilin-1 regulates AMPK-linked signaling pathways.

### 2.2. Synphilin-1 Binds AMPK

**Figure 2:**
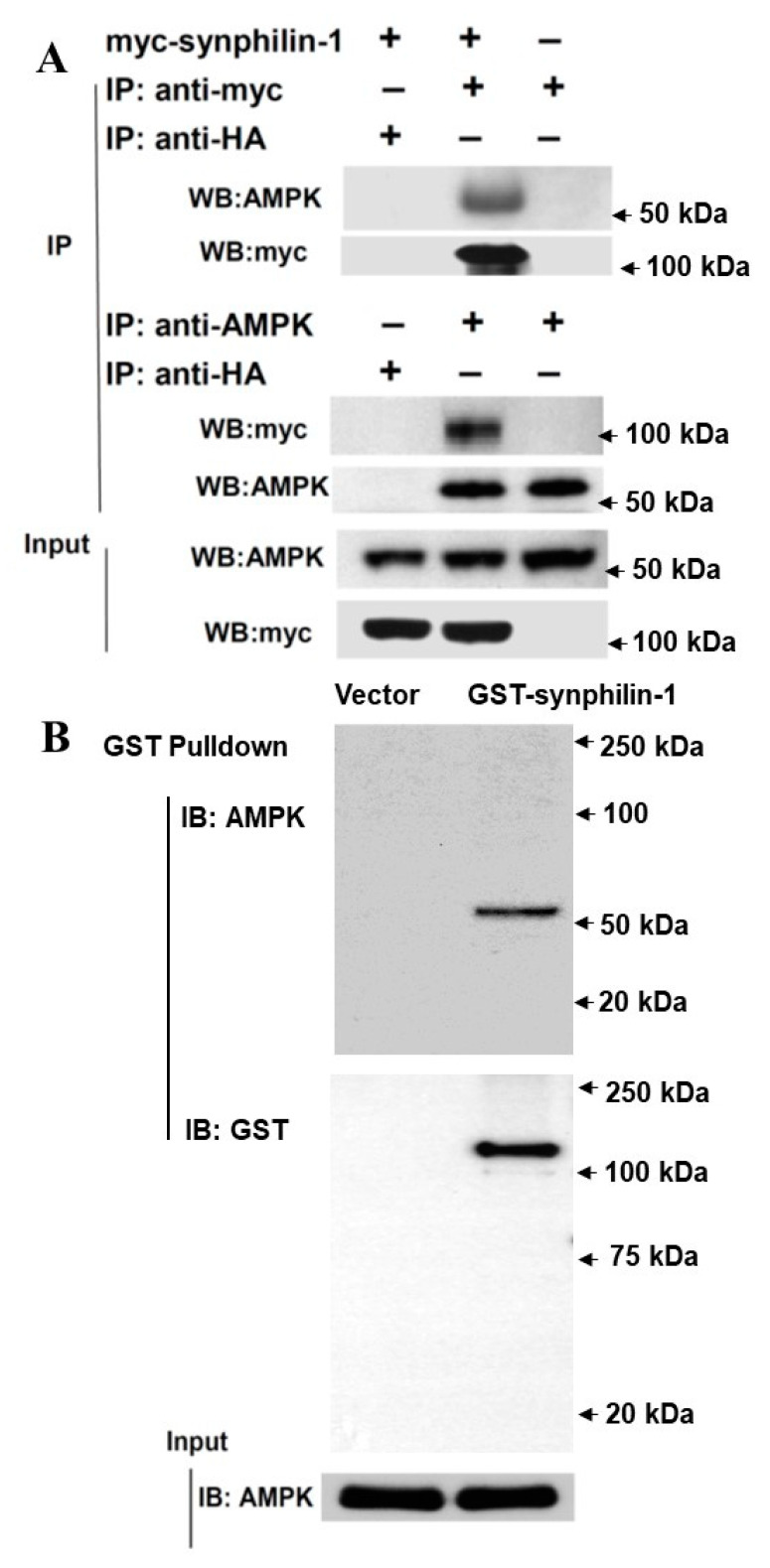
Synphilin-1 interacts with AMPK. (**A**). Lysates prepared from HEK 293 cells transfected with myc-tagged human synphilin-1 cDNA were subjected to IP with anti-myc, anti-HA, or anti-AMPK, followed by anti-AMPK, and anti-myc immunoblotting. Top: Anti-myc antibody precipitated myc-tagged synphilin-1 and AMPK. Middle: Anti-AMPK antibody precipitated AMPK and myc-tagged synphilin-1. Bottom: input blots showing the equal protein loading. (**B**). GST pull-down assays. Top: GST-beads were used to pull-down GST-tagged synphilin-1 and then followed by immunoblotting using anti-AMPK and anti-GST antibodies. Bottom: immunoblotting analysis of input lysates using anti-AMPK antibodies.

To further investigate the relationship between synphilin-1 and AMPK, we tested whether synphilin-1 interacts with AMPK. Synphilin-1 is a cytoplasmic protein and has been shown to interact with other cytoplasmic proteins [7,13,19]. We transfected myc-tagged synphilin-1 into HEK293 cells followed by co-immunoprecipitation assays. Myc-tagged synphilin-1 was immunoprecipitated using anti-myc antibodies, and endogenous AMPK could be detected by anti-AMPK immunoblotting (Figure 2A, top). Conversely, endogenous AMPK was immunoprecipitated using anti-AMPK antibodies, and myc-tagged synphilin-1 could be detected by anti-myc immunoblotting (Figure 2A, middle). To validate this interaction, GST pull-down assays were performed. Pull-down of GST-synphilin-1 also could pull-down AMPK (Figure 2B).

**Figure 3:**
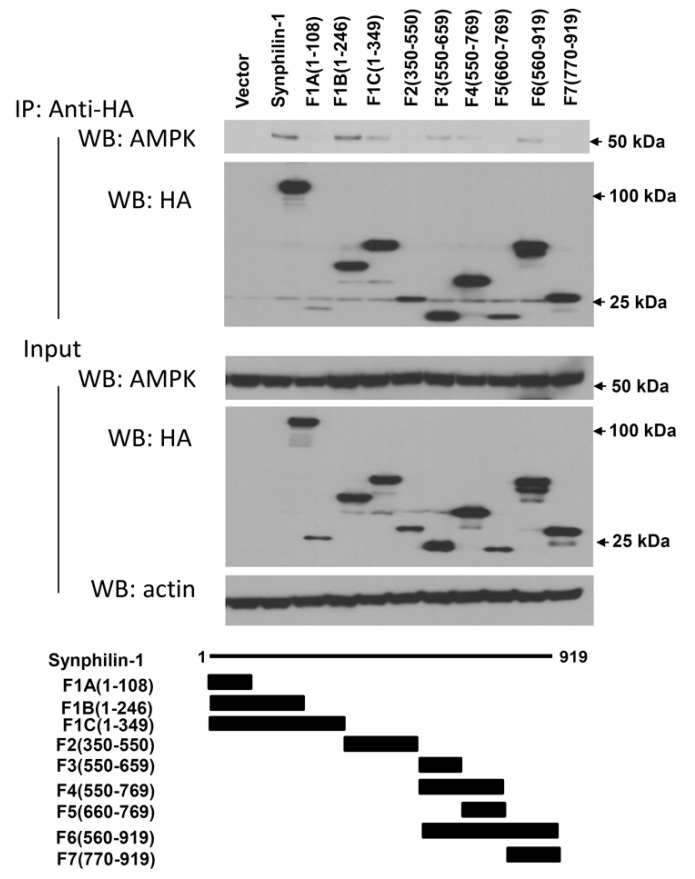
Map of the region of synphilin-1 interacting with AMPK. Lysates prepared from HEK 293 cells transfected with HA-tagged human full length or truncated synphilin-1 constructs were subjected to IP with anti-HA antibody, followed by anti-AMPK and anti-HA immunoblotting. The input of equal protein loading from cell lysates was shown by Western blot using anti-AMPK, anti-synphilin-1, and anti-actin antibodies.

To further map the interaction regions of synphilin-1 with AMPK, HA-tagged truncated synphilin-1 constructs were transfected into HEK 293 cells. The cell lysates were subjected to anti-HA co-IP, followed by Western blot analysis using anti-AMPK antibody. The F1B (1–246 aa), F1C (1–349 aa), F3 (550–659 aa), F4 (550–769 aa), and F6 (550–919 aa) sites interacted with AMPK (Figure 3). In contrast, F1A (1–108 aa), F2 (350–550 aa), and F7 (770–919 aa) did not interact with AMPK. These results indicated that two synphilin-1 regions interact with AMPK: 108–246 aa and 550–769 aa.

### 2.3. Knockdown of Synphilin-1 Reduced AMPK Phosphorylation

**Figure 4:**
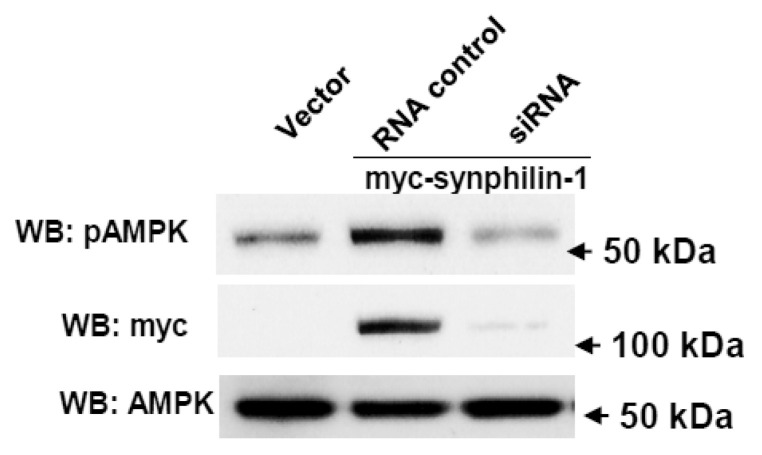
Knockdown of synphilin-1 reduced AMPK phosphorylation. Cells expressing synphilin-1 or vector were transiently transfected with siRNA targeting human synphilin-1 for three days. The cell lysates were subjected to Western blot using anti-myc, anti-phospho-AMPKα, and anti-AMPKα antibodies. Representative blots from three separated experiments.

**Figure 5:**
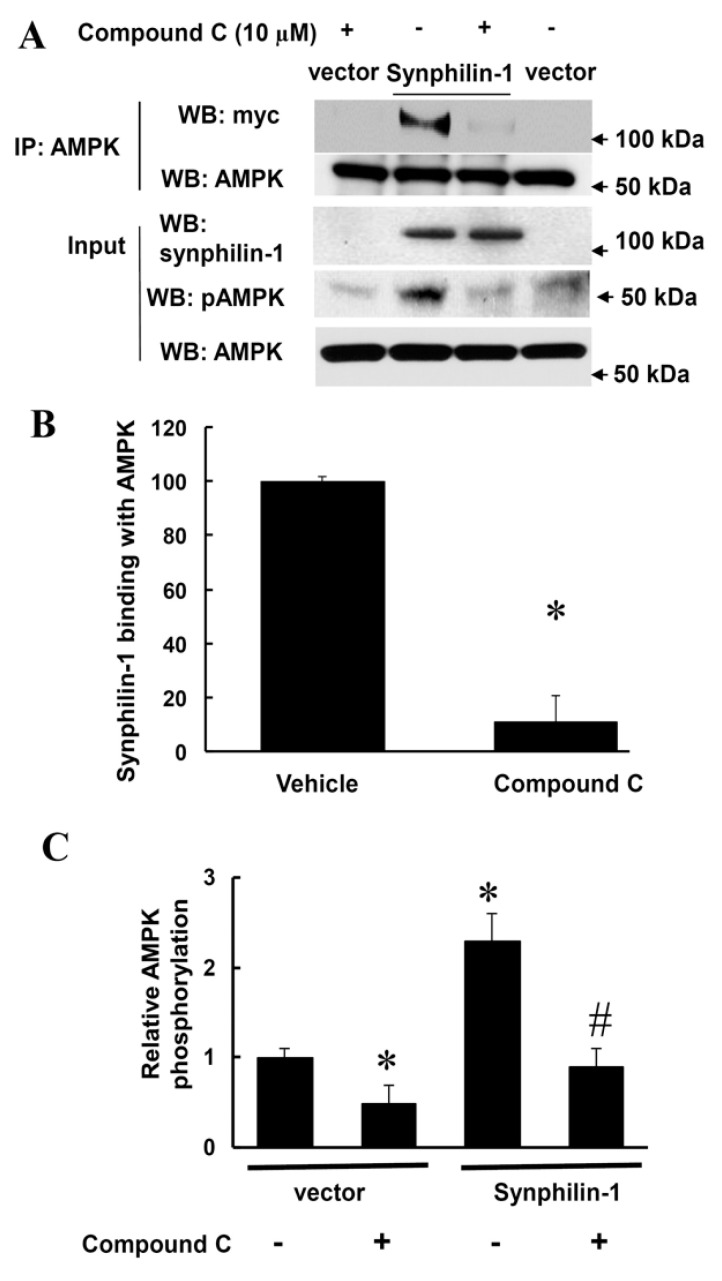
Compound C reduced synphilin-1 binding with AMPK. HEK293 cells were transfected with myc-synphilin-1 and vector, and treated with compound C (10µM) or vehicle for 48 h. Cell lysates were subjected to co-IP using anti-AMPKα antibodies. IP samples and input cell lysates were subjected to Western blot analysis using anti-myc, anti-synphilin-1, anti-phospho-AMPKα, and anti-AMPKα antibodies. (**A**). Representative blots. (**B**). Quantification of synphilin-1 binding with AMPK * *p* < 0.05 by ANOVA followed by Tukey’s post-hoc test, vs. cells expressing synphilin-1 with vehicle treatment. (**C**). Quantification of AMPK phosphorylation levels normalized to total AMPK levels. *p* < 0.05 by ANOVA followed by Tukey’s post-hoc test, * vs. vector cells with vehicle treatment; # vs. cells expressing synphilin-1 with vehicle treatment.

On one hand, reduction of synphilin-1 expression by siRNA significantly attenuated AMPK phosphorylation, compared with cells expressing random control RNA (Figure 4). Whereas on the other hand, treatment with compound C, an AMPK inhibitor, significantly reduced synphilin-1 binding with AMPK (Figure 5A,B). Moreover, compound C reduced synphilin-1-induced AMPK phosphorylation (Figure 5A,C). Our results demonstrated that synphilin-1 mediated AMPK activation, while AMPK activity also regulated the interactions between synphilin-1 and AMPK. These findings suggest that synphilin-1 coupled with AMPK and had interacting effects on each other to regulate cellular activities.

### 2.4. Compound C Attenuated Synphilin-1-Induced Cellular ATP Increase

**Figure 6:**
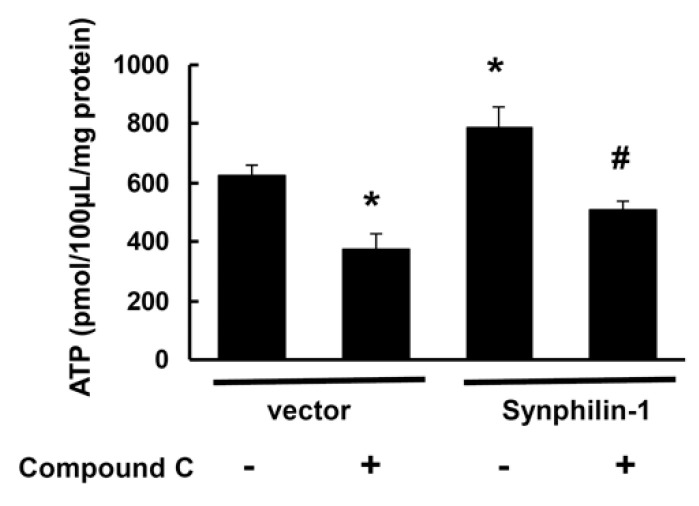
Compound C reduced synphilin-1-induced cellular ATP increase. HEK293 cells were transfected with myc-synphilin-1 and vector and treated with compound C (10µM) or vehicle for 48 h. Cells lysates were subjected to ATP assays. * *p* < 0.05 by ANOVA followed by Tukey’s post-hoc test, vs. vector control cells with vehicle treatment; # *p* < 0.05 by ANOVA, vs. cells expressing synphilin-1 with vehicle treatment.

ATP is the major cellular molecule responsible for providing energy for various cellular processes [4]. AMPK regulates systemic and cellular energy status, and functions as an energy sensor to monitor cellular energy status (ATP levels) in response to nutritional changes [20,21,22,23,24]. Consistent with our previous findings, synphilin-1 increased cellular ATP levels (Figure 6), while treatment with compound C, an AMPK inhibitor (10 µM), attenuated synphilin-1-induced cellular ATP increase (Figure 6). These results indicate that inhibition of AMPK signaling modulates synphilin-1-induced increase in cellular ATP levels.

## 3. Discussion

In this study, we found that synphilin-1 interacted with AMPK, increased AMPK phosphorylation, and resulted in altered activity of the downstream signaling of ACC and p70S6. Knockdown of synphilin-1 reduced AMPK phosphorylation, while inhibition of AMPK by compound C reduced synphilin-1 and AMPK interactions. Moreover, compound C also reduced the amount of synphilin-1-induced AMPK phosphorylation, and cellular ATP increase. These findings indicate that the interaction of synphilin-1 and AMPK play critical roles in regulating cellular energy balance. To our knowledge, this is the first report showing that synphilin-1 alters AMPK signaling, thereby regulating cellular energy status.

Synphilin-1 is a cytoplasmic protein that was cloned over a decade ago [3]. Synphilin-1 was originally identified in interactions with alpha-synuclein and has implications in the pathogenesis of Parkinson’s disease related to protein aggregation and neuroprotection [3,4,7,8,9,19,33]. Recently, we and others have found that overexpression of synphilin-1 increases food intake and body weight in mice [1]. Moreover, synphilin-1 overexpression also results in increases in food intake and body fat storage in flies [2]. In cultured cells, expression of synphilin-1 increases cellular ATP levels [34]. However, the underlying mechanisms of synphilin-1-induced positive energy balance have been unclear. In this study, our results showed that synphilin-1 bound with AMPK predominantly through two regions: 108–246 aa and 550–769 aa. The region 108–246 aa is the ANK-repeat region, which contains a predicted protein–protein interaction domain that has been shown previously to bind with other proteins [3,5,19]. These findings strongly suggest a functional interaction between synphilin-1 and AMPK.

AMPK regulates systemic and cellular energy status, and functions as an energy sensor to monitor cellular energy status in response to nutritional changes [20,21,22,23,24]. AMPK regulates both energy-consuming anabolic pathways and energy-producing catabolic pathways [24,25,35]. AMPK can promote ATP production by increasing nutrient catabolism through regulation of key enzymes at the protein regulation level, while conserving ATP by regulating gene transcription to switch off biosynthetic pathways. ATP is the major energy currency of the cell, and is generated from catabolic pathways (such as fatty acid oxidation and glycolysis) [20,21,22,23,25,35]. ATP provides the energy needed for a variety of cellular activities.

Our results showed that overexpression of synphilin-1 increased AMPK phosphorylation (activation) compared with levels in vector control cells, while knockdown of synphilin-1 attenuated this increase. Moreover, synphilin-1 action on AMPK altered two major downstream targets of the AMPK pathway (ACC and p70S6K) and increased cellular ATP levels. We found that synphilin-1 decreased ACC phosphorylation, in accord with prior reports, which is involved in ATP production by promoting fatty acid oxidation and preventing fatty acid synthesis [25,32]. We also found that synphilin-1 increased the phosphorylation of serine/threonine kinase p70S6K, in accord with prior reports [36,37,38], which is related to maintaining ATP levels by regulating glucose homeostasis and protein synthesis [28], and promoting glucose intake, transport, and glycolytic activity [26]. Our results showed that inhibition of AMPK activity by compound C significantly reduced synphilin-1 binding with AMPK, and attenuated synphilin-1-induced increases in cellular ATP levels. How synphilin-1 activates AMPK remains to be further investigated. One possibility is that synphilin-1 could compete with prolyl-isomerase Pin1 binding AMPK, thereby reducing the interactions between AMPK and Pin1. Previous studies report that Pin1 can bind synphilin-1 and AMPK [33,39]. Pin1 interacts with AMPK and downregulates AMPK activation [39]. Thus, decreased interaction between AMPK and Pin1 and increased interaction between synphilin-1 and Pin1 could result in an increase in AMPK activation. Taken together, these findings indicate that activation of AMPK by synphilin-1 regulates cellular ATP levels.

Synphilin-1-induced AMPK-mediated increases in cellular ATP levels could contribute to two types of cellular activities. One is to provide ample energy for various cellular functions, such as cell growth and protection against toxic stimuli [19]. Previous studies have shown that synphilin-1 has a neurotrophic effect and protects against mutant-α-synuclein and rotenone-induced toxicity in PD models [3,4,7,8,9]. Moreover, increases in cellular ATP levels may also help the sequestration or clearance of toxic proteins by promoting the interactions between synphilin-1 and other proteins (e.g., α-synuclein and parkin) in forming protein aggregates [3,4,7,8,9]. The other potential type of cellular activity of synphilin-1-induced AMPK activation is to augment the energy storage pathways by promoting lipid and glycogen synthesis, which may contribute to fat and glycogen accumulation and deposition [19]. In line with this notion, we previously demonstrated that expression of synphilin-1 induces insulin resistance and increases triglyceride levels and fat storage both in flies and in mice [1,2]. Thus, the increases of cellular ATP levels more likely contribute to the obese-like phenotypes in synphilin-1 transgenic mice and flies [1,2].

In summary, these studies reveal novel cellular functions of synphilin-1 in regulating AMPK-linked signaling pathways, thereby contributing to increases in cellular ATP levels. These findings provide a new molecular basis for understanding synphilin-1 biology and the roles of synphilin-1 in regulating energy homeostasis.

## 4. Materials and Methods

### 4.1. Reagents

Media for cell culture, anti-hemagglutinin (HA) polyclonal antibody, and LipofectAMINE Plus reagent were ordered from Invitrogen (Carlsbad, CA, USA). Anti-AMPKα (AMPK α-subunit), anti-phosphorylated AMPKα (Thr 172), anti-ACCα, anti-phospho-ACCα, anti-p70S6, and anti-phospho-p70S6 antibodies were purchased from Cell Signaling Technology (Beverly, MA, USA). Monoclonal anti-HA was from Roche Molecular Biochemicals (Indianapolis, IN, USA). The anti-myc antibody and the polyclonal anti-HA antibodies were from Santa Cruz Biotechnology (Santa Cruz, CA, USA). The anti-human synphilin-1 polyclonal antibody was generated using the human synphilin-1 fragment (34–500 aa) as the antigen as previously described [3]. Anti-actin antibody was from Sigma (St. Louis, MO, USA). Compound C (an AMPK inhibitor) [40] was from Sigma (Burlington, MA, USA).

### 4.2. Cell Culture, Plasmids, and siRNA Transfection

The myc- or HA-tagged human full-length cDNA are in the pRK5 vector under CMV promoter as described previously [7,13]. Human synphilin-1 fragment constructs were provided by Drs. Christopher A. Ross and S. Engelender [7,13]. Human HEK293 cells were ordered from ATCC and grown in Dulbecco’s modified Eagle’s medium (DMEM; high glucose; Invitrogen) with 10% fetal bovine serum (FBS) and 1% antibiotics (Invitrogen). Transient transfections of various constructs were performed using LipofectAMINE Plus (Invitrogen) according to the manufacturer’s protocol. The specific siRNA targeting synphilin-1 and its scrambled RNA control sequence were purchased from Dharmacon (Chicago, IL, USA). siRNAs were transfected into cells for 72 h using LipofectAMINE 2000 according to the manufacturer’s protocol. Cells were harvested for immunoprecipitation (IP) and Western blot analysis.

### 4.3. Immunoprecipitation (IP), GST Pull-Down and Western Blot Analysis

Cells were harvested in lysis buffer (Cell Signaling) with protease inhibitors (10 µg/mL aprotinin/5 mM PMSF/10 µg/mL pepstatin/10 µg/mL leupeptin) as described previously [16]. The resulting lysates were subjected to IP and Western blot analysis. IP experiments were performed using anti-myc, anti-HA, or anti-AMPK antibodies and protein G Plus/protein A-agarose (GE, Pittsburgh, PA USA) as described previously [7]. GST pull-down was performed as described previously [3]. For Western blot analysis, proteins from each sample were separated on 4–12% NuPAGE Bis-Tris gels, and transferred onto polyvinylidene difluoride membranes (PVDF, Invitrogen) for 3 h. Then the gels were stained with Coomassie blue to assure transfer efficiency. The PVDF membranes were blocked with 5% non-fat milk and incubated with various primary antibodies as listed in Section 4.1 for 1 to 4 h at room temperature. The goat anti-mouse or goat anti-rabbit horseradish-peroxidase conjugated antibodies were used as secondary detection antibodies for 1 h of incubation. Enhanced chemiluminescence reagents (PerkinElmer, Waltham, MA, USA) were incubated for 1 min to detect the proteins in PVDF membranes.

### 4.4. ATP Assay

Cells were harvested in a lysis buffer (Cell Signaling Technology). Lysates were subjected to protein assays using a Bradford protein assay kit (Sigma St. Louis, MO, USA) according to the manufacturer’s protocol. An aliquot of 10 µg of protein from each sample was subjected to ATP luminescent assays using an ATP Determination Kit (Invitrogen) according to the manufacturer’s protocol. The experiments were repeated three times in duplicate.

### 4.5. Data Analysis

Quantitative data were represented as means ±Standard Error of the Mean (SEM). Protein and ATP levels were used for one-way or two-way analysis of variance (ANOVA) by Sigmastat 3.1 statistical software (Aspire Software International, Leesburg, VA, USA). Post-ANOVA analysis of group differences was performed with the Tukey test. A *p* value < 0.05 was considered significant.

## Data sharing statement

The data that support the findings of this study are all included in this manuscript.
